# Observed regression of rapidly growing multifocal squamous cell carcinoma of the face and scalp following initiation of doxycycline

**DOI:** 10.1016/j.jdcr.2026.07.059

**Published:** 2026-08-04

**Authors:** Raj P. Fadadu, Samantha N. Scerbo, Laura S. Romero

**Affiliations:** University of California, San Diego Department of Dermatology, San Diego, California

**Keywords:** doxycycline, neoplasm, oncology, skin cancer, squamous cell carcinoma, tetracycline, tumor regression

## Introduction

Rapidly growing, multifocal cutaneous tumors in an adult warrant a thorough evaluation to distinguish between infectious and neoplastic etiologies, particularly when lesions are numerous or progressive. This assessment should include careful clinical history, targeted systemic work-up, and tissue sampling for histopathology, immunohistochemistry, and microbiology cultures.

Spontaneous regression of cancer has been reported in several malignant cutaneous tumors, including keratoacanthoma, squamous cell carcinoma (SCC), basal cell carcinoma, melanoma, Merkel cell carcinoma, cutaneous metastases, certain lymphoproliferative neoplasms, and Kaposi sarcoma.^1^^,^^2^ We present a case of rapidly progressing multifocal cutaneous squamous cell carcinoma followed by tumor regression after initiation of oral doxycycline, raising the possibility that doxycycline may have acted alongside host immunity and microenvironmental factors to promote tumor regression.

## Case report

A 79-year-old man presented to dermatology clinic with rapidly growing skin lesions on his left face, scalp, and left hand causing discomfort for the past 4 weeks. He denied any localized trauma, recent travel, or exposure to sick contacts. Review of systems was negative for systemic symptoms like fevers, chills, cough, joint pain, and diarrhea. His medical history was notable for treated basal cell carcinomas of the face and squamous cell carcinomas of the extremities. He was taking no immunosuppressive medications. He frequently gardens and had prior exposure to alcohol and tobacco over many years as well as Agent Orange while serving in the military.

**Fig 1:**
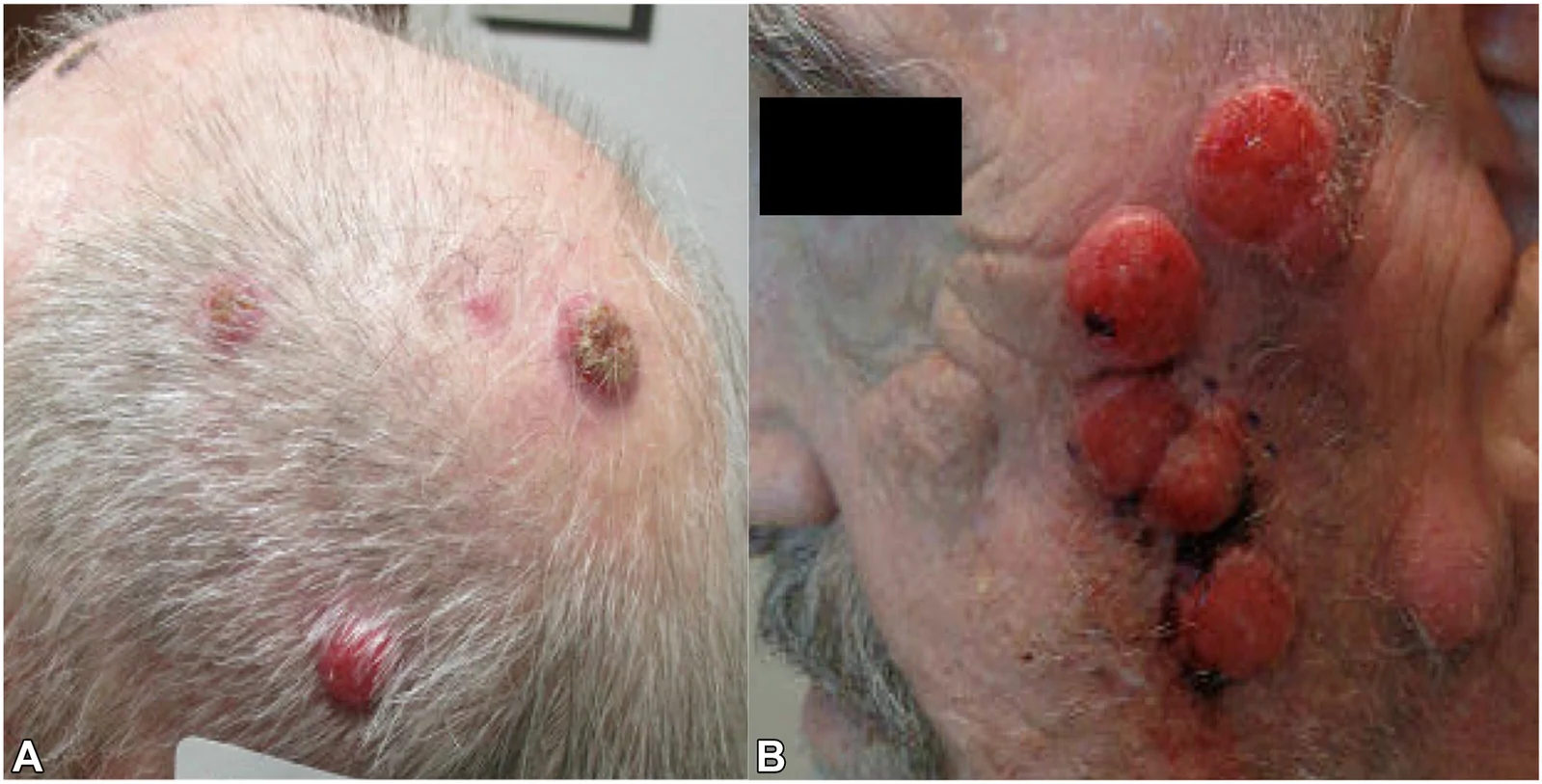
Initial presentation showing extensive nodular lesions on the **(A)** scalp and **(B)** left lateral face.

The physical exam demonstrated 6 clustered exophytic eroded nodules (1.5-2.5 cm) on the left side of the face, 6 scattered shiny erythematous nodules (1-1.5 cm) on the scalp with yellow crust, and 2 erythematous nodules (0.75 cm) on the left hand (Fig 1). He had no cervical, supraclavicular, or axillary lymphadenopathy. Based on the clinical presentation, the differential diagnosis was broad and primarily including neoplastic and infectious etiologies, such as deep fungal skin infection, atypical mycobacterial infection, multifocal squamous cell carcinoma, eruptive keratoacanthomas, cutaneous metastases, and cutaneous lymphoma.

A comprehensive diagnostic evaluation was performed to assess for infectious and malignant causes. The work up was largely unremarkable, aside from a superficial bacterial skin swab positive for *Staphylococcus aureus*. Testing for human immunodeficiency virus, syphilis, tuberculosis, aspergillosis, blastomycosis, histoplasmosis, and coccidioidomycosis, as well as fungal and mycobacterial tissue cultures, was negative. Computed tomography of the head demonstrated masses confined to the soft tissue without bony invasion, and computed tomography of the chest/abdomen/pelvis showed no evidence of internal malignancy or metastatic disease.

**Fig 2:**
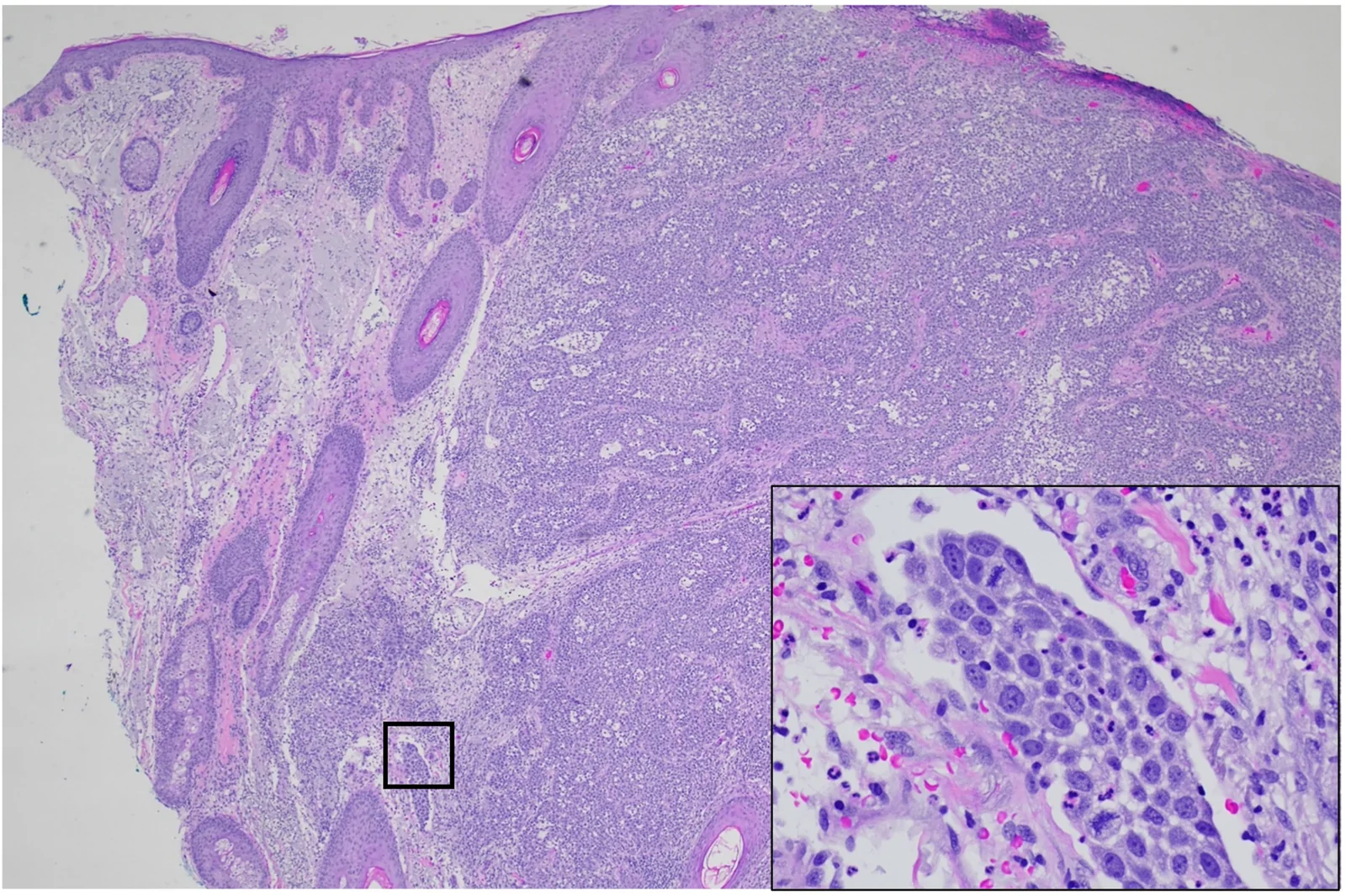
Left cheek skin histology, H&E, ×2 magnification; inset: H&E, ×40 magnification showing lymphovascular invasion.

**Fig 3:**
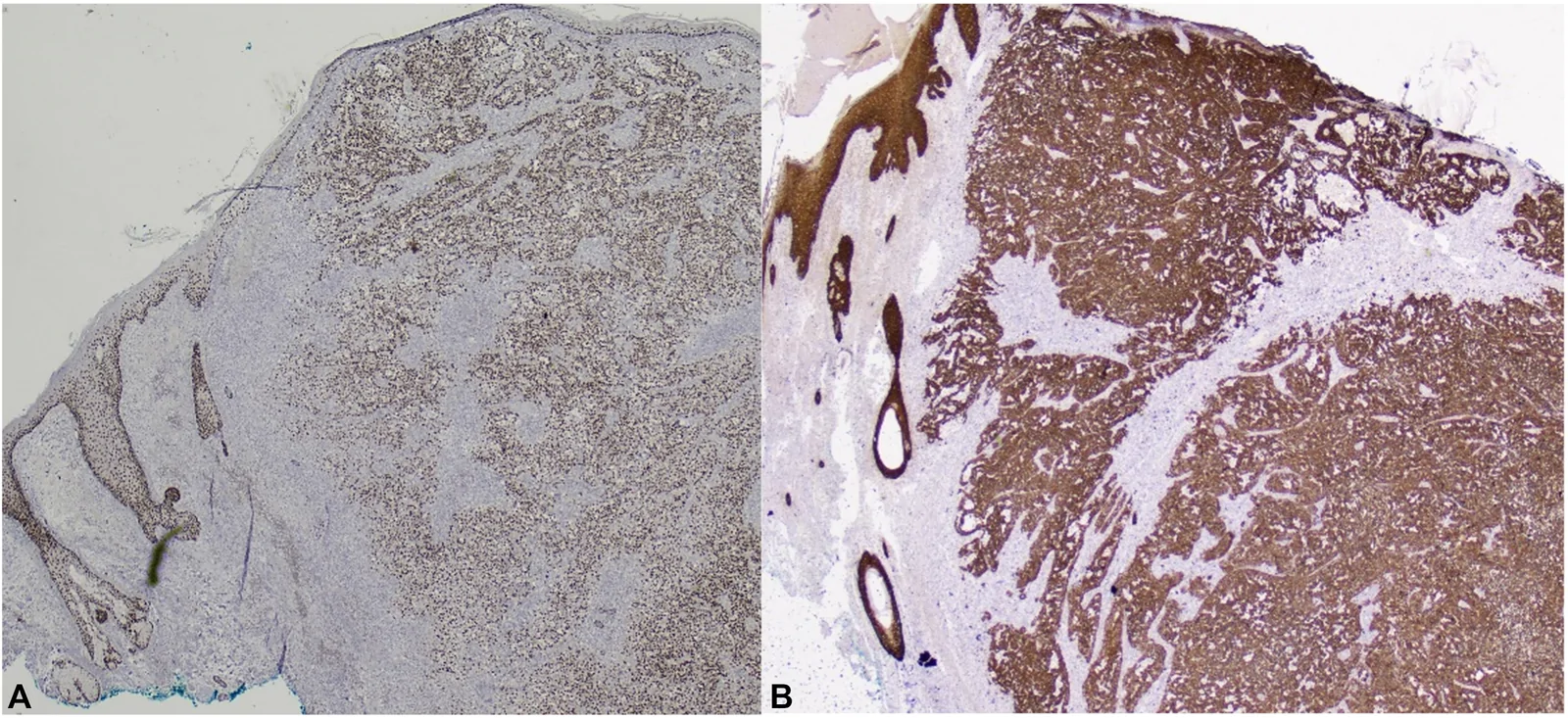
Immunohistochemistry stains for **(A)** p63 and **(B)** cytokeratin 5 demonstrate positivity from skin biopsy.

Punch biopsies were obtained from 2 representative lesions on the scalp and cheek only; the remaining 12 lesions were not sampled. Dermatopathology evaluation demonstrated identical findings consisting of a poorly differentiated epithelioid tumor with clear cell features. Lymphovascular invasion was absent in the scalp lesion but present in the cheek specimen (Fig 2). Immunohistochemistry showed positive results for p63, GATA3, and CK5 (Fig 3) and negative results for androgen receptor, adipophilin, CD30, CD45, calretinin, SOX10, CK7, CK20, PAX8, and NKX3.1. Next generation sequencing was performed on a skin sample, which showed a high tumor mutation burden of 18.9 mutations/megabase and the following mutations: PIK3CA (8.5% variant allele frequency), CDKN2A, ASXL1, PTEN, HRAS, ARID1A, and TP53. In combination, these results supported a diagnosis of multifocal squamous cell carcinoma.

**Fig 4:**
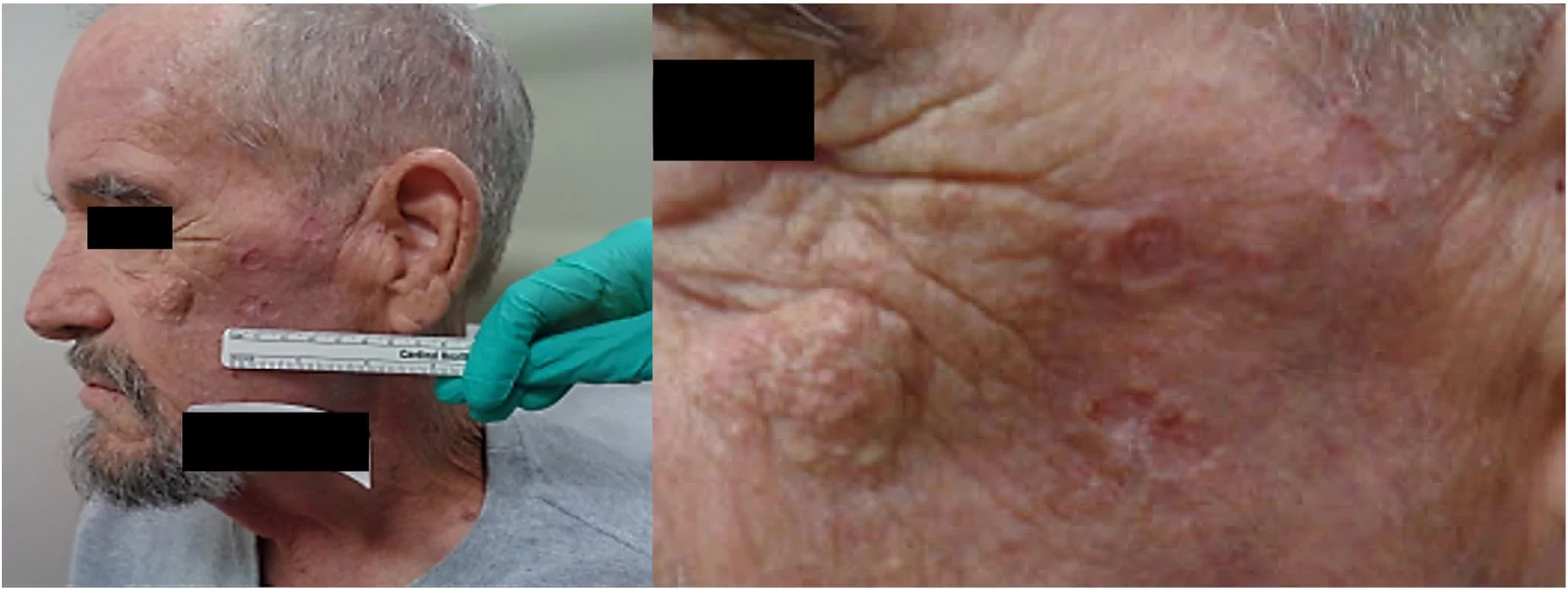
Updated clinical photos showing resolution of tumors with atrophic scarring 4 months after initial presentation.

The patient was started empirically on doxycycline 100 mg twice daily due to the crusted appearance of the lesions at presentation, and therapy was continued after cultures grew *Staphylococcus* species. The aforementioned work-up was completed over several weeks, during which the patient was seen periodically in clinic. Demonstrable regression was noted at each visit, and doxycycline was actively continued as the only notable intervention timed with this regression. Although surgical intervention was initially considered, serial follow-up examinations demonstrated progressive, concurrent regression of all nodules, both biopsied and nonbiopsied. Complete clinical resolution with atrophic scarring was observed at 4 months (Fig 4). No lesions were re-biopsied during the regression phase, so histologic confirmation of regression was not obtained. Doxycycline was discontinued 1 month later. There has been no recurrence of these tumors in the following 16 months during serial clinical evaluation with full-body examination, lymph node assessment, and review of systems. A position emission tomography/computed tomography scan obtained 10 months after initial presentation noted no increased cutaneous or lymph node activity but did find a hypermetabolic solitary right lung nodule that is being further evaluated.

## Discussion

Several biological mechanisms have been proposed to explain spontaneous tumor regression, particularly in cutaneous SCC. Immune-mediated tumor destruction is thought to play a central role, with activation of CD4^+^ and CD8^+^ T cells and a Th1-predominant cytokine response, including interferon-γ signaling, contributing to tumor involution. Reports of spontaneous regression of cutaneous SCC and cutaneous metastases describe prominent lymphocytic infiltrates and heightened local immune activation, supporting the importance of host immune surveillance.^3^, ^4^, ^5^ Regression has also been observed following partial diagnostic biopsy, suggesting that tumor antigen exposure or local tissue injury may stimulate a secondary immune response capable of promoting tumor clearance.^5^ In the present case, partial biopsy of 2 representative tumors could have contributed to regression, although regression of the other 12 nonbiopsied lesions argues against biopsy-induced local antigen exposure as the sole driver for regression. Cases of regression following cessation of immunomodulatory therapy, such as ruxolitinib, further underscore the role of immune restoration in tumor control.^6^ Additional contributing factors may include alterations in the tumor microenvironment, including antiangiogenic signaling and stromal changes that limit tumor growth and survival.

The potential contribution of doxycycline to tumor regression also warrants consideration. Beyond their antimicrobial effects, tetracycline-class antibiotics have demonstrated anti-inflammatory and antineoplastic properties in preclinical models.^7^ Proposed mechanisms include inhibition of matrix metalloproteinases, modulation of mitochondrial function, interference with cancer stem cell activity, and effects on epithelial–mesenchymal transition and tumor-associated inflammation.^7^ In SCC specifically, doxycycline has been shown in vitro and in vivo to downregulate matrix metalloproteinase expression, reduce cellular migration and invasion, and suppress tumor growth in xenograft models.^8^ Given the established association between increased matrix metalloproteinase expression and SCC invasion and metastasis, these findings provide biologic plausibility for a potential adjunctive antitumor effect.

While a direct causal relationship cannot be established in this case, the temporal association between doxycycline initiation and tumor regression raises the possibility that antibiotic-related antitumor effects, immune modulation, or an interaction between the 2 may have contributed to the observed clinical course in this patient. Overall, this case adds to the limited literature describing regression in cutaneous SCC and highlights the complex interplay between tumor biology, host immunity, and potential medication effects. Further investigation into the immunologic and pharmacologic mechanisms underlying spontaneous regression may help clarify whether tetracycline class antibiotics could have therapeutic relevance in selected cases of cutaneous malignancy.

### Declaration of generative AI and AI-assisted technologies in the writing process

There was no use of artificial intelligence related to this article.

## Conflicts of interest

Dr Fadadu reports receiving honorarium from L'Oreal Dermatological Beauty for Advisory Board work unrelated to the submitted work. Dr Romero and Author Scerbo have no conflicts of interest to declare.
